# European digital transformation for Roma girls’ health: the case of Spain

**DOI:** 10.3389/fsoc.2025.1562088

**Published:** 2025-07-22

**Authors:** Daniela E. Miranda, Valeria Terán-Tinedo, João Henrique Borges Bento, Tomas de Jong, Jaisalmer de Frutos-Lucas

**Affiliations:** ^1^Department of Social Psychology, Center for Community Research and Action (CESPYD), University of Seville, Seville, Spain; ^2^European Public Health Alliance, Brussels, Belgium; ^3^School of Social Sciences and Communications, Universidad Europea, Villaviciosa de Odon, Madrid, Spain

**Keywords:** Roma women, digital divide, digital determinants of health, digital rights, social justice, digital transformation, adolescence

## Abstract

The digital experiences of Roma girls are shaped through the Digital Determinants of Health (DDoH) such as poor housing conditions with limited digital infrastructure, under-resourced public schools in marginalized neighborhoods, and dominant narratives that overlook their capacities and contributions. Although the European digital transformation advances rapidly, it often fails to address the enduring impacts of structural antigypsyism across DDoH. This article addresses the following questions: (1) how do European digital transformation efforts respond to the impacts of historical antigypsyism on Roma girls’ digital experiences? (2) What dynamics across societal, community, interpersonal, and individual levels shape the violation and protection of Roma girls’ digital rights in marginalized neighborhoods? and (3) How can European digital transformation policies be operationalized to support a shift from impoverished to meaningful digital participation for Roma girls across all levels of the digital ecosystem? To answer these, the study employs a triangulation approach integrating policy analysis of relevant European strategies on digitalization and Roma inclusion, field observations from ongoing participatory research, and a targeted literature review providing theoretical and comparative grounding. The triangulation of these sources supports the development of an analytical framework for Roma Girls’ Digital Rights that allows to map digital experiences through an ecological lens. By bridging policies with local realities, this paper provides actionable recommendations to operationalize macro-level policies with the everyday realities of Roma girls, ensuring that they can experience digitalization as a pathway to empowerment and as active contributors to Europe’s digital future.

## Introduction

Roma girls across Europe are growing up on the digital margins as a result of historical antigypsyism (Digital Freedom Fund, n.d.). Antigypsyism has been defined as a historically persistent form of racism against Roma, characterized by homogenizing perceptions, attributing deviant traits, and discriminatory social structures that degrade, ostracize, and perpetuate structural disadvantages (Alliance Against Antigypsyism, 2016). Roma girls face compounded disadvantages shaped by their ethnicity, age, class, and gender. These intersecting factors, underpinned by antigypsyism, have long-term effects on their health and well-being. The negative impacts on Roma girls’ health from childhood to adulthood, evidenced through data related to child health (e.g., respiratory pathologies, low immunization), poor housing conditions, unemployment and lower life expectancy (Fernández-Feito et al., 2019). This reality now manifests in the digital realm, as the digital transformation has spearheaded initiatives across health and social systems that pose new risks to Roma girls’ health (Garmendia and Karrera, 2019; WHO, 2022). This was evident during the COVID-19 pandemic, when government regulations limited in-person interactions during the state of emergency assumed a baseline level of digital capacity in Spanish households–such as consistent internet access, laptops or tablets, and digital literacy to navigate online platforms (Jupîneanţ et al., 2024). The regulations disrupted in-person schooling, shifted access to social benefits online with new technical requirements. However, the regulations failed to account for the lack of digital infrastructures of Roma families living in marginalized contexts (Albar-Marin et al., 2022). The pandemic and post pandemic Europe revealed societies’ greater reliance on digital tools and its risks and opportunities related to public health.

The European digital transformation has been framed as a promising avenue to rectify widening disparities. In its “Path to the Digital Decade” Policy Programme (European Commission, 2021), Europe has proposed to carry out a digital transformation of public services and economy to ensure the EU’s digital leadership and promote human-centred, inclusive and sustainable digital policies that empower citizens. As a result, the European Parliament, the Council and the Commission (European Parliament and Council of the European Union, 2022a) have established common values, rights and principles for the digital decade, outlining commitments in key areas such as universal connectivity, digital education, data protection, and fairness in the digital and labor environments. It also advocates for the responsible use of Artificial Intelligence (AI) and algorithm, and emphasizes the protection of children and youth in digital spaces. Through its commitment to “a digital transformation that leaves nobody behind,” the declaration on digital rights seems to represent an opportunity for marginalized communities since it holds transformative potential to improve their health and wellbeing, empower them to pursue their aspirations, and allow them to make informed choices in the digital environment. In this sense, digital transformation has shown to be able to empower marginalized communities and offer new avenues for self-sovereignty and agency (Qureshi, 2022), although it requires ensuring meaningful inclusion and addressing the digital divide (Datta and Thomas, 2021; Mehra et al., 2004; Oreglia, 2014).

This paper aims to analyze the relationship between EU digitalization policies and the current contexts of marginalized neighborhoods in Seville, Spain to understand the ways forward in operationalizing EU goals in local contexts. The study aims to contribute to the growing field of digital health in relation to Roma communities. Three main research questions are addressed: (1) How do European digital transformation efforts respond to the impacts of historical antigypsyism on Roma girls’ digital experiences?, (2) what factors across societal, community, interpersonal, and individual levels shape the violation and protection of Roma girls’ digital rights in marginalized neighborhoods, and (3) how can European digital transformation policies be operationalized to move Roma girls’ experiences from impoverished to meaningful digital participation across all levels of the digital ecosystem?

The next section proposes an analytical framework to map the digital experiences of Roma Girls’ to understand the impact of policies and practice at the local level.

### Roma girls’ digital experiences: an analytical framework

Emerging research since the COVID-19 pandemic revealed that Roma communities across Europe experience a digital divide, especially young Roma women (Albar-Marin et al., 2022; Velicu et al., 2022). The digital divide refers to inequalities in access to and use of Information and Communication Technology (ICTs), which stems from structural disparities such as limited access to infrastructure, economic constraints, and educational barriers (Peláez-Sánchez and Glasserman-Morales, 2023), leading to broader social inequalities that impact individuals’ rights and quality of life (Reinsalu, 2022). In this line, digital literacies and internet connectivity have been identified as the “super social determinants of health,” since they address all other social determinants of health (Sieck et al., 2021). Research shows that digital engagement mediates access to healthcare, social benefits, and employment (Heponiemi et al., 2024) but also determines the digital footprints in which data-driven systems make decisions (Allmann and Radu, 2023). However, the notion of digital poverty extends the discussion from access and skills to the quality of engagement with digital technologies. This concept is particularly relevant for Roma communities, as it encompasses not only technological barriers but also how cultural exclusion and systemic biases prevent meaningful digital participation, which hinder individuals and groups from benefiting from digitalization to improve their quality of life (Digital Poverty Alliance, 2022; The British Academy Digital Society, 2022).

Richardson et al. (2022) refers to the Digital Determinants of Health (DDoH) as conditions that impact a variety of risks and outcomes related to health, functioning, and quality of life, that operate at the individual, interpersonal, community, and societal levels of influence. The DDoH are based on the concept of the social determinants of health (Fraser et al., 2023). This is defined by the WHO as “the non-medical factors that influence health outcomes*”* such as our education, employment or the place where we live (WHO, n.d.).

Chidambaram et al. (2024) identify nine dimensions of DDoH, namely, ease of use, usefulness, interactivity, digital literacy, digital accessibility, digital availability, digital affordability, algorithmic bias, technology personalization, and data poverty and information asymmetry. These digital determinants show how the adoption of new technologies can affect healthcare utilization and access, and in certain situations, exacerbate pre-existing sociodemographic disparities that have an additional effect on health outcomes such as quality education and employment opportunities. In this paper, the term ‘*digital experiences*’ will refer to the daily experiences within the digital transformation shaped by the DDoH.

**Figure 1:**
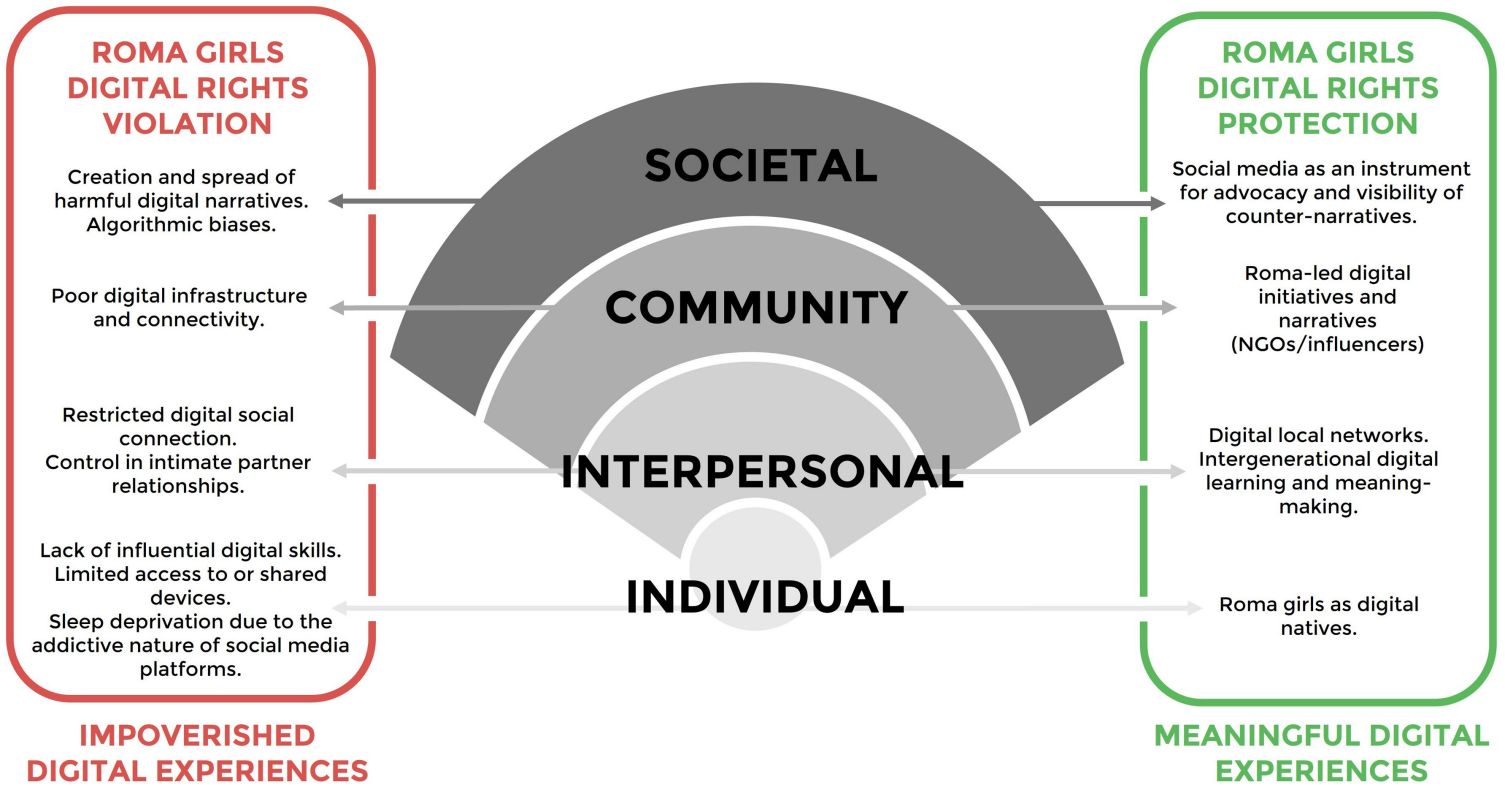
Analytical framework for Roma girls’ digital rights.

Digital experiences range from impoverished digital experiences to meaningful digital experiences that reflect either the violation or protection of digital rights. Digital rights encompass the freedom and protections that individuals are entitled to in digital spaces, such as their right to privacy, data protection, freedom of expression and other emergent rights in the digital era (Chidambaram et al., 2024; Custers, 2022; Hutt, 2015; Pangrazio and Sefton-Green, 2021). As it is represented in Figure 1, the violation of digital rights exacerbates existing Roma health inequities mediated through impoverished digital experiences. Conversely, digital rights protection fosters Roma girls’ health and wellbeing, which reflects meaningful digital experiences, or the capacity of digitalization to empower Roma girls through equitable access to opportunities and the creation of environments where their digital rights are safeguarded. Digital rights protection can develop a sense of recognition, agency, and inclusion for Roma Girls as represented in the digital determinants framework.

**Figure 2:**
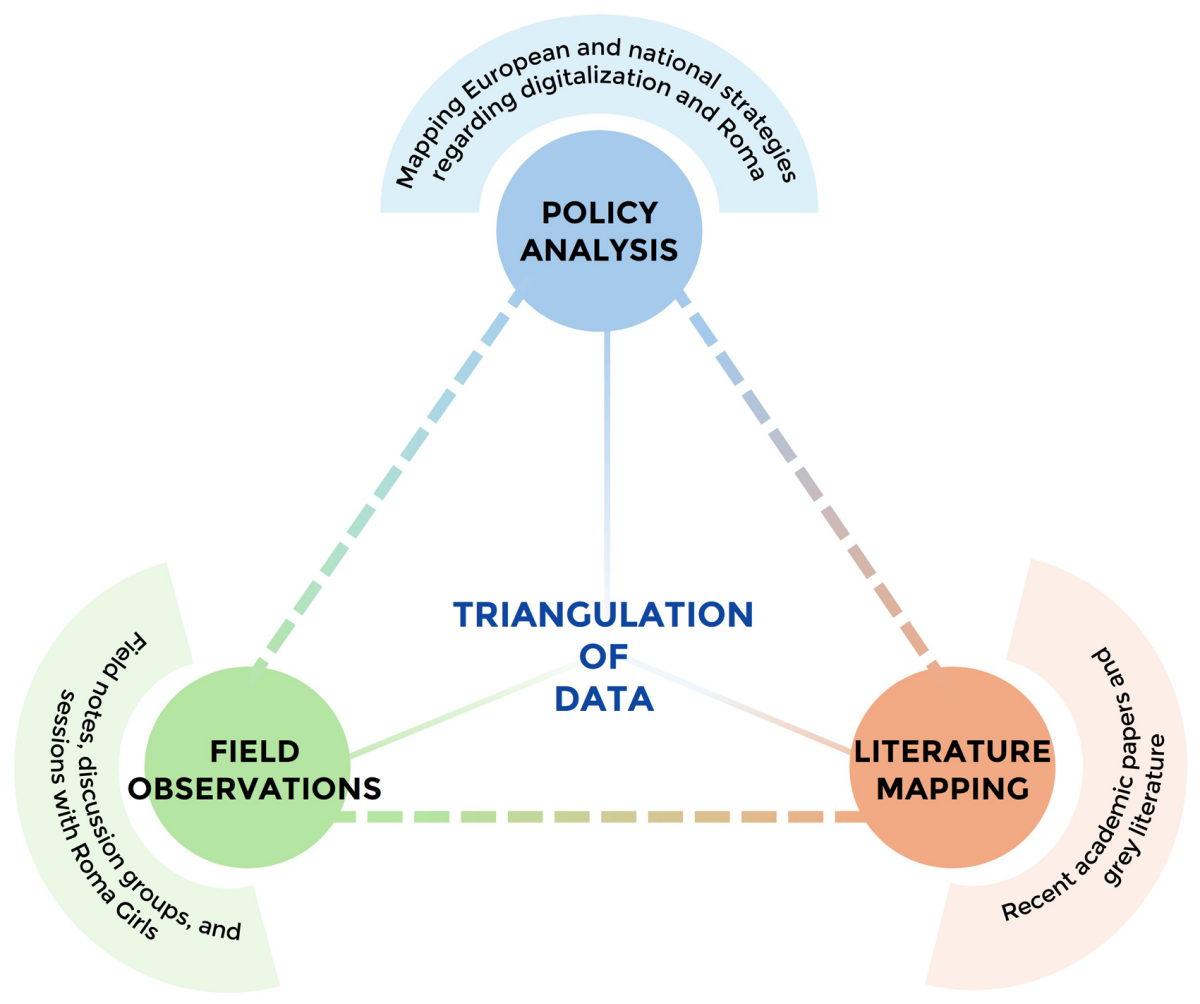
Policy and practice review triangulation.

The societal level encompasses structural, political, and cultural forces, such as policies, economic systems, and social narratives, that indirectly dictate the digital experiences of Roma girls by either reinforcing or challenging systemic antigypsyism. Systemic antigypsyism is perpetuated by deeply embedded social norms that reinforce the exclusion of Roma Girls from mainstream society. One critical manifestation of this discrimination involves the perception of Roma as lacking the capability or competence to meaningfully participate in decision-making processes, which lead to their voices being consistently excluded from policy development. The absence of Roma perspectives in these spaces ensures that their concerns remain invisible to policymakers, thus creating digital policies that neglect their interests and concerns. Consequently, this contributes to the development and perpetuation of algorithmic biases that manifest when existing human biases become embedded in training datasets that underrepresent or misrepresent Roma girls, or when trained systems engage in discriminatory decision-making that systematically disadvantage Roma in different domains (e.g., employment, health, housing). Protecting their digital rights requires structural changes such as including regulation of algorithmic fairness, development of inclusive digital policies relevant for Roma communities, and advocacy efforts to amplify Roma girls’ voices in policy-making processes, ensuring that their perspectives are reflected in the digital transformation (see Figure 2).

At the community level, barriers such as inadequate community infrastructure, healthcare systems that do not integrate digital tools, and exclusionary community norms perpetuate digital inequality. Violations at this level may result in Roma girls being disconnected from the digital environment. Collective efforts such as digital inclusion programs and local advocacy initiatives can dismantle barriers to digital participation, allowing Roma girls to take advantage of the opportunities offered by digitalization. At an interpersonal level, digital rights violations occur when implicit biases in Roma girls’ social circles discourage them from accessing and utilizing digital resources. Protecting their digital rights at this level entails building supportive networks of family, peers, and mentors that can create and/or promote safe spaces where Roma girls feel encouraged to explore and benefit from digital tools. At the individual level, the violation of Roma Girls’ Digital Rights often manifests as limited access to technology, poor digital literacy, and a lack of confidence in navigating digital tools. Roma girls facing these barriers are excluded from educational, social, and economic opportunities offered by digital transformation. On the other hand, digital rights protection involves digital self-efficacy, ensuring digital literacy and access to affordable technology for Roma girls to meaningfully engage in digital spaces.

Understanding the complexity of digital rights involves identifying the multi-level elements for developing effective interventions and policies. Within the Spanish context, translating EU-level policies into actionable recommendations requires careful examination of how these dynamics manifest from policy development to local implementation in neighborhoods that have been historically marginalized to promote Roma girls’ meaningful digital experiences.

### Policy and practice review approach

In order to map the digital experiences of Roma girls, a triangulation approach was employed integrating: (1) *policy analysis* to review the relevant European and national policies on Roma inclusion and digitalization to establish the broader policy context. (2) *field observations* were conducted in educational settings within the Torreblanca and Polígono Sur neighborhoods, where researchers gathered detailed notes on lived experiences of Roma girls such as the daily interactions and practices related to digitalization. These observational insights were organized and interpreted through the lens of the ecological model, which allowed us to contextualize individual, interpersonal, community, and structural factors; (3) Complementing these data, a targeted *literature review* provided theoretical grounding and comparative perspectives with the observational insights. This triangulation of multiple sources enabled a nuanced understanding of the interplay between policy frameworks, lived experiences, and scholarly knowledge, enhancing the depth and validity of this analysis.

## Policy analysis

### European digital policy landscape

Emerging European policies aim to regulate the digitalization of public services across European countries. The EU Digital Strategy (European Commission, n.d.-a) is key, a comprehensive plan that aims to shape Europe’s digital future, fostering the use of technology to enhance economic growth, improve public services, and empower citizens; while ensuring inclusion, sustainability and safety. The EU Digital Strategy includes initiatives such as the *Artificial Intelligence Act* (European Parliament and Council of the European Union, 2024), the *Digital Services Act* (European Parliament and Council of the European Union, 2022a), the *Digital Markets Act* (European Parliament and Council of the European Union, 2022b), and the *European Health Data Space* (European Commission, n.d.-b), currently the most ambitious health-related EU policy project. These regulations aim to provide personalized, efficient and accessible health and social care, opportunities for building equitable futures and improve secure health-data sharing. Ultimately, this would contribute to reducing disparities across social determinants of health. Yet, little evidence portrays how these regulations transform the realities in the neighborhoods of Roma girls living in marginalization, especially in relationship to key determinants such as employment, civic participation and educational outcomes that influence health outcomes. These initiatives are based on the assumptions of reliable digital connectivity and a set of digital competences.

For example, the AI Act (European Parliament and Council of the European Union, 2024) requires AI systems to provide transparency, accuracy, accountability and clear information about how decisions are made. However, to ensure these goals are reached it is not sufficient to request that AI systems developers provide this information, it is essential that individuals are equipped with the necessary skills to critically assess the information provided by AI systems and healthcare professionals about the use of AI (Alowais et al., 2023). Similarly, the recent agreement for creating and regulating the European Health Data Space (EHDS) poses an additional challenge for marginalized communities. According to the original proposal, this framework aims to “*empower individuals to have control over their health data*” (European Commission, n.d.-b). Empowering citizens requires sufficient levels of digital literacy and equitable access to digital tools to fully understand the risks and benefits of data sharing and provide informed consent when necessary to align with AI Act. However, this again assumes that educational settings, communities and housing have the necessary digital infrastructures and resources to foster critical digital skills.

Moreover, the Digital Services Act (DSA) (European Parliament and Council of the European Union, 2022a) explicitly mentions that extensive online platforms and search engines should deploy the necessary means to mitigate systemic risks, where there is “*foreseeable negative effect on the protection of public health, minors and serious negative consequences to a person’s physical and mental well-being, or on gender-based violence.”* Targeted marketing techniques have the potential to exert a negative influence on health, when they effectively influence individuals to consume unhealthy products, raising a public health concern (Grier and Kumanyika, 2010). Accordingly, the DSA prohibits targeted advertising based on sensitive data (e.g., ethnicity, religion, or sexual orientation), which intends to protect marginalized groups from exploitative or discriminatory practices. Nevertheless, social media platforms can exploit ambiguities in these definitions and can also manipulate personal data in various ways (e.g., aggregating or inferring data, relying on user behavior or third-party partnerships) that enable them to bypass these prohibitions. These indirect user practices have been attributed to a “deep information asymmetry” that requires better regulation (Smith, 2023).

All these examples demonstrate the impact of the digital determinants of health. In a rapidly digitalizing world, access to health and wellbeing is mediated by access to digital literacy, skills, devices, and infrastructure within a just context. Limited interaction with digital services due to a lack of skills or access can further undermine the health of marginalized communities that become invisible in datasets, and therefore also for policymakers or AI system developers (Allmann and Radu, 2023). Inclusive and evidence-informed policy calls for a better understanding of the underlying factors that lead to digital inequities and therefore health inequities, or in the words of Davies et al. (2021): address the digital inverse care law. The inverse care law states that those individuals who already experience health inequities most, and those with greater health needs, are also the least likely to have access to, or engage with, technology. In this sense, according to the World Health Organization 2023 report *Digital Health in the European Region*, while 83% of Member States have a national digital health policy and/or strategy, only 52% have developed digital health literacy policies and/or strategies. For instance, in the case of Spain, the Ministry of Health (2021) aims to empower citizens to take care of their health. Still, it fails to provide a roadmap to improve the responsiveness of digital services to Roma people that respond to the multiple factors that affect their health and well-being.

The EU Digital Decade Targets 2030 (European Commission, 2024) is an initiative that seeks to overcome the digital divide by ensuring for instance that, by the year 2030, 80% of adults in the EU have basic digital skills, every household has access to gigabyte-speed internet, and digital devices are safe to use and affordable. This initiative also aims to achieve the complete digitalization of public services, where 100% of key public services in the EU are expected to be available online and 100% of citizens will have access to a digital identity and online medical records. It is still unknown how these measures will impact Roma adolescent girls living in marginalized neighbourhoods, but the 2030 targets seem insufficient to achieve their digital inclusion without addressing historical antigypsyism. For example, there is no operationalised definition of basic digital skills to critically assess whether these will be sufficient to safely navigate the digital world that the 2030 targets pursue. Additionally, 20% of adults are estimated to lack these basic digital skills, which according to the digital inverse care law are more likely to be individuals with already poorer health and experiencing social exclusion (Davies et al., 2021). Finally, even if digital identities and health records are granted to all citizens, 5% percent of the population in Europe (24 M approximately) are non-EU citizens (where Spain presents the second highest percentage) (Eurostat, 2023) and up to 1% (4-5 M) are estimated to represent silent ranks–such as undocumented migrants, stateless people and people of unknown citizenship, a much higher number than the 356,000 reported by the European Commission (Eurostat, 2023; Connor and Passel, 2019). Therefore, ensuring the digital inclusion of all individuals surely requires a more comprehensive approach and a wider view.

### Bridging digitalization policies to Roma, child and health policies

Efforts to realize digital inclusion can be strengthened through digital policy, but the causes for inequities do not lie exclusively within the digital policy sphere. A 2022 World Health Organization report, titled ‘*Equity within digital health technology within the WHO European Region*’, found that too little attention is paid to the more complex drivers and systems that impact digital health literacy. In fact, solutions to drive digital health inclusion must consider factors such as existing health conditions, population characteristics, and the health services themselves (WHO, 2022).

When exploring digital inclusion in Europe along these social lines, there is evidence that suggests there are digital inequities, especially along lines of age, gender, and rurality. Nevertheless, digital health exclusion is rarely considered, despite the risk this poses to increasing health inequities (Woolley et al., 2023). These digital health considerations should be integrated across national and EU equality strategies. Subsequently, when talking about the inclusion of key populations, the EU’s Union of Equality Strategies form the designated policy instrument. To illustrate, the *EU Anti-racism Action Plan 2020–2025* explicitly mentions that the digital transition provides an opportunity to fight against racism, but can also provide risks if not used or framed correctly (European Commission, 2020a). Similarly, the *EU Roma Strategic Framework 2020–2030* points to the importance of ensuring digital inclusion to realize socio-economic inclusion (European Commission, 2020b). Furthermore, the *Spanish National Roma Strategic Framework* underlined the urgency of action on digital inclusion, citing disparities in access based on low income, literacy and education levels in Spain in 2020. For example, 84.5% of those earning below €900 lacking connectivity compared to 99% of higher earners, and only 51.4% of illiterate individuals connected, with Roma more often represented in these populations (Ministerio de Derechos Sociales y Agenda 2030, 2020).

Another relevant policy instrument is the *European Child Guarantee*, which includes a focus on the digital divide, encourages member states to improve access to digital infrastructure and to develop digital skills (Council of the European Union, 2021). Consequently, the *Spanish Child Guarantee Action Plan 2022–2030* sets objective 2.6 as “*end the digital divide*,” focusing particularly on children who live in poverty or are socially excluded. Progress is measured according to indicators including internet access and devices.

Aside from the sociocultural environment, another domain to be addressed is the health care system itself. One challenge is that health care systems and the digital tools used therein are a national competence, and therefore a public good. Even so, the EU has policy instruments that are relevant to both encouraging inclusive health care systems, and to mainstream digital inclusion. Through the *European Semester*, input can be provided into priority setting in the budgetary plans of member states, including on social policy (European Commission, n.d.-c). This creates opportunities to promote investment in inclusive health systems, as well as digital health tools. Similarly, the European Commission contributes to mainstreaming social policy and inclusion across its activities through the *European Pillar of Social Rights (ESPR)* and the *EPSR Action Plan*. This spans different sectors, but also specific actions on digital development, such as the *Digital Education Action Plan 2021–2027* and the aforementioned *Digital Decade 2030* (European Commission, n.d.-d). Only by embedding digital health considerations across these cross-cutting policy frameworks can long-term solutions be achieved; focusing solely on digital policy would be insufficient.

The following section zooms in to the local level in Seville, Spain—a city that reflects the challenges and opportunities of implementing EU-level digital strategies in practice.

## Field observations and evidence mapping

Despite being part of a broader European digital landscape, Seville is home to two of the poorest neighborhoods in the country, Polígono Sur and Torreblanca. Polígono Sur, located in the southern part of Seville, encompasses several neighborhoods, including Las Tres Mil Viviendas, and has an estimated population ranging between 30,000 and 35,000 residents (Jiménez Barca, 2024). Developed in the late 1960s and early 1970s to accommodate populations relocated from informal settlements, Polígono Sur is home to a significant number of Roma families, many of whom experience severe and overlapping forms of socioeconomic exclusion that include low household income, high unemployment, limited access to quality education and healthcare, and housing precarity. Basic utilities such as water and electricity are often unstable or illegally accessed, and many dwellings are in poor structural condition. The neighborhood also faces serious public safety concerns related to organized crime, leading to insecurity, extortion, and forced displacement of residents (de Sevilla, 2018).

Torreblanca, a peripheral neighborhood in Seville, is divided into *Torreblanca la Vieja, Las Lumbreras* and *Torreblanca la Nueva*. The latter, developed in the early 1960s, was established through public housing initiatives such as the *Patronato de Casas Baratas* to accommodate families displaced from informal settlements. Today, *Torreblanca la Nueva* houses the largest population density in the neighborhood, with Roma families residing primarily in small, overcrowded public housing units. This area is marked by significant structural disadvantage, including high unemployment, educational exclusion, and poor infrastructure, leading to its classification as a zone with “Special Needs for Social Transformation” (de Sevilla, 2018). Miranda et al. (2022) conducted a community-based participatory action research project where local participants documented environmental health hazards, such as unsanitary conditions and neglected public services, highlighting the normalization of substandard living conditions and the absence of Roma representation in community decision-making spaces.

These neighborhoods offer a stark contrast to the aspirations of the EU Digital Strategy, revealing how systemic barriers and localized inequities can hinder the realization of policy goals. By examining the digital experiences of Roma girls and their communities in this context, and linking it to existing literature, the authors aim to outline a potential roadmap for a rights-based digital transformation.

Emerging findings from ongoing projects in Spain by the Center for Community Research and Action at the University of Seville in partnership with Asociación YILÓ, GAZ KALÓ, and La Fragua Projects—ANDAROMI, ROMACARE and NEXTROM are presented. These initiatives operate at both national and local levels. ANDAROMI-ROMACARE, funded by the Andalusian Regional Government, explore the role of Roma women and girls in care-related work and the recognition of their contributions to societal advancement, with a focus on fostering intergenerational partnerships to innovate within social services through entrepreneurial endeavors and increased digital competences. These projects are currently being implemented in three high schools (ages ranging from 13 to 16 years old) and two elementary schools (ages range from 9 to 12 years old) located in Torreblanca and Polígono Sur, two neighborhoods characterized by high levels of socioeconomic exclusion (Agencia Tributaria, 2022). In parallel, the NEXTROM project, financed by the Spanish Ministry of Science, Innovation and Universities, seeks to identify gaps and opportunities for digitalization within the European and National Roma Strategic Frameworks. At the intersection of these three projects, the authors have gathered the emerging insights through discussion groups involving Roma-led organizations, Roma community leaders, Roma adolescent girls, educators, academics, and regional and local policymakers.

In the following section, emerging insights from these projects, daily observations from researchers in these neighborhoods, are organized and compared to existing literature to provide a comprehensive understanding of digital transformation and its impact on Roma girls.

To complement and deepen the insights gained from field observations, scientific and grey documents (eg. third sector reports) were mapped, focusing on digital experiences of Roma and non-Roma groups that share similar structural discrimination. This mapping exercise allowed to situate the observational data within broader academic and policy discussions, facilitating a comparison between lived experiences and existing knowledge. The observational data and literature are organized based on levels defined in Richardson et al. (2022) within the analytical framework proposed.

### Societal

Social media platforms are defined as commercial determinants of health, or private sectors prioritizing profit over health outcomes, paradoxically allowing for increased youth participation while targeting advertising and the spread of misinformation (Pitt et al., 2024). These platforms are based on algorithms designed to maximize engagement, tending to amplify sensational and exclusionary content. These dynamics have concrete consequences: for example, YouTube, TikTok and other social media outlets are amplifying harmful content about Roma communities, exposing Roma youth at a young age to stigmatizing narratives reinforced and propagated by algorithmic systems (Breazu and Machin, 2023).

Recent research suggests that youth of color exposed to persistent online discriminatory content shape how they perceive themselves and their wider community (Bravo et al., 2019; Tao and Fisher, 2022). Additionally, these harmful narratives have been shown to cause significant psychological distress in youth, such as depressive symptoms (Wachs et al., 2022). In neighborhoods like Polígono Sur in Seville, social media and policing have developed a new dynamic in which viral TikTok videos featuring police raids and neighbors’ reactions exploit elements associated with Roma culture. Growing up in the shadow of these portrayals impacts Roma girls’ identity formation, just as offline antigypsyism has been shown to do (Willem and Gonçalves, 2022).

The persistence and amplification of such narratives stems from the design of algorithmic systems that privilege discriminatory content. Algorithms that power social media platforms, lending services, and recruitment processes sometimes inherit existing antigypsyism biases (Noble, 2018; Eubanks, 2018). A recent example demonstrates how these biases also manifest in generative AI tools that depend on existing data and content. In November 2024, the first and second author (DM and VT) prompted ChatGPT to “create an image that reflects Roma women in Europe.” The output capitalized on tropes, failing to represent the realities and experiences of Roma women and girls accurately. This incident underscores the biased data embedded in open source AI tools, which rely on incomplete datasets when utilizing text-to-image features (De Caleya Vázquez and Garrido-Merchán, 2024).

Digital discrimination through harmful narratives, coupled with algorithmic biases, creates a cycle of marginalization that continues to perpetuate systemic inequities in the digital sphere. For instance, hashtags linked to Polígono Sur reinforce the digital footprint of Roma communities within the framework of antigypsyism. Yet there are examples of the power of social media to share counter narratives. In October 2024, social media served as a critical tool for condemning police brutality during an operation in Polígono Sur, following a major shootout in the area. These events highlight the importance of digital platforms, as they are instruments for visibility and defense for marginalized communities.

Other examples show promising avenues for social media platforms to undo historical harms. One illustration of these efforts to foster empowerment and drive social change through digital storytelling comes from Manuel Jiménez, a video blogger based in Seville’s Polígono Sur neighborhood. Polígono Sur is often depicted as the less favorable side of Seville. Through short TikTok videos highlighting neighborhood customs, family life, and the strong community bonds that characterize the area, he offers a dignified, positive portrayal that challenges prevailing stereotypes (García, 2024).

### Community

Community spaces (e.g., schools, community centers) and homes are not able to provide stable digital connectivity due to other political actors such as utility companies. Both neighborhoods have long battled with the national electric utility, ENDESA, S. A., over improving energy infrastructure. Grassroots movements and public debates have consistently highlighted the risks posed by poor infrastructure, particularly regarding the conservation of foods or for those with chronic illness that depend on external electrical devices to sustain health (Europa Press, 2024). Rhetoric associating neighborhoods like Polígono Sur with illegal activities, such as drug plantations, has only exacerbated this neglect–as reflected in the societal level section. Now, the lack of these infrastructures or accountability can impact a wider range of resources too due to the need of these services to have digital connectivity. McCall et al. (2022) describe this phenomenon as “digital redlining,” where low-income communities face systematic reductions in broadband services and digital access. In Polígono Sur and Torreblanca, families often rely on non-governmental organizations to help access digital connectivity and digital certifications needed for welfare benefits such as basic minimal income. This reliance on intermediaries maintains the hierarchical relationships between organizations-communities to access goods through digital resources.

The challenges with internet infrastructure are evident in the daily implementation of projects like ANDAROMI-ROMACARE, which take place in elementary and high schools, and weekly meetings in a community center. The research team frequently encounters difficulties accessing Wi-Fi, often relying on personal mobile data to respond to these immediate needs. Again, this creates a hierarchy in which university-based personnel have access to reliable resources, while community partners, including Roma girls, face significant limitations in accessing digital tools and materials. University-based partners bridge these gaps by sharing digital materials and teaching the use of tools, such as ChatGPT and app-related services (e.g., Uber) with community partners, or developing online maps and surveys with Roma adolescent girls (e.g., Typeform). Despite these anecdotal efforts, this reflects an on-going issue where Roma living in marginalized contexts rely on external resources which do not provide a long-term, sustainable and empowering response.

The digital exclusion at the community-level will affect Roma girls over the life course. In adulthood, Roma women rely on informal economic sectors like market vending and recycling. Digital transformation in the educational system remains disconnected from the employment realities of Roma communities, failing to provide pathways for these girls to participate in a digital economy. Evidence from the NEXTROM discussions revealed that there are no policies that protect Roma current entrepreneurship endeavors, and there is a risk digitalization–such as online transfer or e-commerce–will increase control of Roma’s form of resistance to an economy that has traditionally excluded them. Roma-led organizations are at the forefront in the quest for digital inclusion in terms of employment. Organizations such as the Fundación Secretariado Gitano have developed programs (Training for a Digital Future; Aula Digital) aimed at improving digital literacy and skills among Roma youth. These initiatives arm Roma youth with tools for education and employment while offering a safe space for peer learning and empowerment.

### Interpersonal

Garmendia and Karrera (2019) observe that Roma adolescents, like their non-Roma peers, use digital spaces primarily to coordinate and share offline activities with friends and family. However, for Roma girls, digital engagement remains predominantly rooted in their immediate social spheres. Several studies (Correa, 2015; Abu-Asbah, 2018; Juliana and Anshori, 2023) underscore the generational gaps in how technology is accessed and used, a problem that is especially significant in Roma families. In many cases, it is Roma girls who end up teaching their families how to use technology—especially smartphones, which are often the only available devices (Munté-Pascual et al., 2025; Fundación Secretariado Gitano, 2023). By doing this, these young women help boost digital know-how, narrow the generational divide, and strengthen the family bond. At the same time, their families’ limited digital literacy can put these teenagers at risk, since research shows how important adult guidance is for safe and beneficial technology use among children and young people (Garmendia et al., 2016). From the research team observation, Roma girls engage with digital platforms like TikTok as a form of self-expression and recreational activity, with access to homogenous content that is reinforced by the shared meaning and utility of these platforms in their surroundings.

Finally, according to community partners’ observations, some Roma girls are overwhelmed with the control that their partner’s have from a young age using mobile location tracking, calling into question the ways in which sharing location can be used as a form of control between intimate partner relationships especially in these contexts. Whether it was shared social media profiles, access and sharing passwords, and controlling the communication flow, issues around privacy are closely linked to girls’ agency. Research shows that mobile phones are a double-edged sword that in a sense can provide young women with control over relationship formation, but can also be a means of control in intimate partner relationships (Gibbs et al., 2021). A deeper exploration is required regarding how Roma girls’ mediate their understanding of relationship formation through these digital mechanisms.

### Individual

Roma teenagers are currently growing up in the digital era, being considered part of a generation of “digital natives,” that is, people who have grown up in an environment surrounded by technologies and who have greater familiarity with their use and access (Prensky, 2001). According to the Fundación Secretariado Gitano (2023), about nine in ten Roma households had smartphones. Around 78% had internet at home, 31.9% owned a tablet, and 22.3% had a computer. Roma girls are growing up as digital natives; however, there is a significant difference between simply being immersed in a digital environment and possessing robust digital skills (Li and Ranieri, 2010). While teenage girls are surrounded by technology, they often encounter barriers that prevent them from fully benefiting from the opportunities digitalization can provide. These challenges are frequently rooted in limited digital literacy, low access to devices, and unreliable internet connections (Garmendia and Karrera, 2019). Digital literacy has today become that indispensable skill, without which no child and young person would be able to meaningfully engage with online education, formal and informal learning; acquire access to life-saving information on health and well-being; find community and be able to leverage economic, employment, and entrepreneurship opportunities (Meherali et al., 2021).

While working on the ANDAROMI-ROMACARE project, the researchers noticed that smartphones were in constant use—mostly for scrolling through social media applications like TikTok, which has recently become extremely popular for its short video clips. The research team has identified a recurring reality: many girls report feeling exhausted at school and falling asleep during classes due to disrupted sleep patterns caused by late-night TikTok use. Sleep deprivation during adolescence can have long-term consequences, including increased mental distress, physical health challenges and disruptions to daily life (Kelly et al., 2018). Educators and specialists often attribute this problem to the girls’ habits or to parental shortcomings. However, critical discussions with community organizations highlight broader systemic factors, such as the lack of resources available to the girls competing with the addictive nature of TikTok, which research has shown to disproportionately affect adolescents by offering a sense of connectivity.

## Discussion

This paper aimed to contrast how digital policies are framed and then translated into the realities of Roma girls at the local level. The analytical framework identifies the digital determinants of health (DDoH) that impact the digital experiences of Roma girls. This insight defines actionable recommendations across societal, community, interpersonal and individual levels described in the following section.

### Actionable recommendations for a fair Roma digital transformation

At a *societal level*, involving Roma girls in developing and regulating algorithms, drafting accessible policies, and uplifting the voices of Roma girls are all steps toward a digital future that is truly equitable. From the design of educational curricula to the development of digital tools—including AI generative applications—it is crucial to integrate the perspectives and aspirations of Roma girls. To address these issues and to make sure that algorithms benefit everyone fairly, it is of utmost importance for public policies to ensure transparency, equity, and impartiality in how they operate. Routine audits and well-defined standards for algorithmic justice help prevent digital platforms from mirroring or deepening existing inequalities (Binns, 2018; Selbst et al., 2019). The AI Act ensures that high-risk AI systems, such as those used in education or recruitment, adhere to principles of fairness and non-discrimination, reducing the potential for biased algorithms to exacerbate existing inequalities. A standard for meaningful digital experiences under the AI Act could include algorithms that harness counternarratives, such as the video blogger in Polígono Sur that denounced police brutality, demonstrating their potential role in advancing institutional accountability and addressing specific, localized digital determinants of health. Interdisciplinary partnerships among government agencies, human rights groups, tech companies, researchers and Roma communities is just as important as making sure that Roma girls have a seat at the table. Through these collaborations, targeted initiatives can address digital challenges in ways that reflect local realities and nuances (European Roma Rights Centre, 2022). This would ensure that local authorities foster public engagement and implement accountability measures that address the digital determinants of health.

Civil society groups play a key role in the digital ecosystem of Roma girls by pushing a critical approach to algorithmic governance. Researchers observed how local civic society organizations made significant efforts in retweeting and centering Roma influencers to reach a broader audience. Civil society actors can go a step further, for example, like the AlgoRace movement^1^ that aims to center racialized voices in AI governance and deployment. In 2024 FAKALI, a Roma-led umbrella organization launched AmalIA, an AI tool offering interactive games and educational content on Roma history and culture, illustrating how Roma identity continues to evolve in the digital realm (FAKALI, n.d.). Roma girls could potentially acquire economic independence through digital entrepreneurship. If Roma girls were provided assistance to develop the right digital set of skills, ensured they had the proper tools and showed them how to start learning e-commerce, these girls could reach new audiences to strengthen their financial status (Girl Effect, 2020).

Societal level dynamics can be reflected in the daily occurrences at the *community level*. Increasing the meaningful digital experiences of Roma girls requires educational spaces to shift their focus, recognizing and valuing the capacities and interests of Roma girls. While Roma girls exhibit strong capabilities with platforms like TikTok, integrating such tools into educational activities that align with their needs remains a challenge. Based on the observations within the context of the fieldwork reflect the misalignment between EU goals and the digital infrastructures available in schools and community centers. Educators in these spaces are managing multiple challenges and harnessing digitalization will require local authorities to adapt resources and incentives for both educators and the wider community. Extracurricular offerings for Roma girls are often limited to care-related training (e.g., esthetician or hairdresser), without a forward-looking vision that includes digital skills development. Regional education offices, in collaboration with Roma civil society organizations, should expand these offerings to include coding workshops, digital storytelling, and AI literacy, all rooted in relevant pedagogies. Schools and educators must be equipped to address the complexities of antigypsyism by adhering to EU digital policies while advocating for Roma-sensitive mechanisms for their implementation (Munté-Pascual et al., 2025). The EU Digital Education Action Plan promotes cooperation between Member States and the exchange of good practices. In this context, it is important that the experiences of projects that have successfully worked with marginalized communities are taken into account and that Member States can adapt digital education policies to the specific contexts and demands of Roma girls. Finally, current school debates around phone bans and technology use must be examined through the lens of digital determinants of health and digital rights. National and regional ministries of education should develop inclusive guidelines that ensure Roma girls are not excluded from beneficial digital experiences, and that technology access is framed as a right essential to their development, autonomy, and future opportunities.

The community-level infrastructures and opportunities for digital engagement influence the *interpersonal level*, where digital experiences intersect with family dynamics and daily contexts. Regarding intimate relationships, Roma girls are protected under the EU Digital Services Act, which is aimed at creating a safer, more transparent, and accountable online environment, including provisions for the protection of minors online. Under this regulation, platforms are expected to take appropriate measures to ensure a high level of privacy, safety, and security for minors. Roma girls often use platforms like TikTok from a young age to connect, communicate, and engage with social references, including influencers that resonate with their identities. While this provides opportunities for expression and learning, it also exposes them to risks that require tailored protections. Under the EU Digital Services Act (EU, 2022), online platforms are obligated to implement measures that protect minors, including ensuring privacy, safety, and age-appropriate content moderation. However, enforcement must be contextually grounded. Local authorities, think tanks, private and public partnerships could collaborate with Roma-led organizations and educators to create targeted campaigns and guidelines that promote safe practices aligned with digital rights in platforms widely used by Roma girls. This in turn could provide schools with tailored content to embed in their syllabi.

The observations at the local level found that Roma women and girls were using social media and accessing digital resources together. Schools, universities, civil society organizations and families can co-develop intergenerational digital literacy programs that bring together Roma girls and older Roma women. These partnerships could take the form of family-based workshops, peer-to-peer mentoring, or digital journalism projects that re-dress those mentioned in the societal issues above (For digital storytelling see: www.otherfrontline.org). Digital journalism has the potential to elevate the lived experiences of Roma women and girls to advocate for actions oriented towards the DDoH on a community and societal level. These programs would foster a shared understanding of online safety within the wider communities. By grounding digital safety in existing interpersonal relationships these efforts ensure that Roma girls can fully benefit from digital spaces without compromising their wellbeing or autonomy.

The interconnected dynamics at each level land on the *individual level*, the focus must be put on the development of critical digital skills to navigate digital environments safely and effectively. The challenge lies in threading digital literacy interventions with existing digital capabilities, educational objectives and Roma girls’ community values. Current policies may not account for the structural barriers that accumulate over the life course of Roma women and girls. The exclusion from digital tools at a young age prevents Roma girls from acquiring key digital competencies that European policies emphasize for labor market transitions and future employment within the “Path to the Digital Decade” Policy Programme (European Commission, 2021). The aim is to increase the employability of Roma girls through revaluing their current knowledge and aspirations, and connecting them to current challenges related to the economy of care while increasing their digital competences along the way. Partnerships between civil society organizations and the private sector can support Roma girls as digital advocates while learning transferrable digital skills. Local employment services and vocational training programs should develop targeted schemes that tie digital learning to emerging care economies.

## Conclusion

This policy and practice review contributes to the field in three significant ways. First, it specifically centers on Roma girls’ experiences within digital transformation processes, addressing a notable gap in the literature that has largely overlooked the gendered dimensions of digital marginalization within Roma communities. Second, it proposes an analytical framework for Roma girls’ digital rights that connects European digital policies to local realities, providing an ecological lens to understand policy implementation gaps. Third, it advances both Roma studies and digital Roma studies by moving beyond descriptive accounts of digital exclusion to propose actionable recommendations for policy operationalization at multiple scales. By developing and applying a framework focused specifically on Roma girls, the study contributes to Roma knowledge production with both theoretical insight and practical guidance for ensuring digital transformation functions as a tool for empowerment, not further marginalization.

The disconnect identified in this analysis suggests the importance of developing mechanisms to systematically monitor how digital policies affect vulnerable populations, especially Roma communities, across member states and candidate countries. Opportunities for future research can build on the analytical framework for Roma girls’ digital rights developed in this study. This could enhance understanding of which contextual factors are most influential in shaping meaningful digital experiences while outlining common patterns of digital marginalization. Further, translating these insights into practical change could benefit from co-design methodologies that enable local actors to assess and enhance digital inclusion efforts in their specific contexts, creating solutions for continuous feedback between policy design and local realities. Algorithmic justice research could also build upon this study to examine more deeply how automated decision-making systems impact Roma communities, particularly girls. As algorithmic systems increasingly influence access to essential services across healthcare, education, and social welfare domains, understanding how these technologies might perpetuate or disrupt patterns of antigypsyism becomes crucial for ensuring that digital transformation serves as a tool for equity. In this sense, Roma girls should be recognized as innovators related within the digital transformation, and political actors in health and care systems. It is vital to spotlight digital rights so that Roma communities know how to defend themselves against discrimination and abuse online (Amnesty International, 2021). The development of technologies should follow a logic that addresses historical antigypsyism by involving Roma women and girls in their creation and deployment. Seville, home to major political actors and evaluators in Artificial Intelligence, offers opportunities to mobilize influential local partnerships. To ensure a fair European digital transformation, the lived experiences of groups whose digital rights are violated by historical injustices must be at the forefront of policy and practice.

## Footnotes

^1^ https://www.algorace.org/informate/
